# Verbal autopsy of 80,000 adult deaths in Tamilnadu, South India

**DOI:** 10.1186/1471-2458-4-47

**Published:** 2004-10-15

**Authors:** Vendhan Gajalakshmi, Richard Peto

**Affiliations:** 1 Epidemiological Research Center, Chennai, India; 2 Clinical Trial Service Unit & Epidemiological Studies Unit (CTSU), University of Oxford, Oxford, UK

## Abstract

**Background:**

Registration of the fact of death is almost complete in the city of Chennai and not so in the rural Villupuram district in Tamilnadu, India. The cause of death is often inadequately recorded on the death certificate in developing countries like India. A special verbal autopsy (VA) study of 48 000 adult (aged ≥ 25 yrs) deaths in the city of Chennai (urban) during 1995–97 and 32 000 in rural Villupuram during 1997–98 was conducted to arrive at the probable underlying cause of death to estimate cause specific mortality.

**Methods:**

A ten day training on writing verbal autopsy (VA) report for adult deaths was given to non-medical graduates with at least 15 years of formal education. They interviewed surviving spouse/close associates of the deceased to write a verbal autopsy report in local language (Tamil) on the complaints, symptoms, signs, duration and treatment details of illness prior to death. Each report was reviewed centrally by two physicians independently. Random re-interviewing of 5% of the VA reports was done to check the reliability and reproducibility of the VA report. The validity of VA diagnosis was assessed only for cancer deaths.

**Results:**

Verbal autopsy reduced the proportion of deaths attributed to unspecified and unknown causes from 54% to 23% (p < 0.0001) in urban and from 41% to 26% (p < 0.0001) in rural areas in Tamilnadu for adult deaths (≥ 25). The sensitivity of VA to identify cancer was 95% in the age group 25–69.

**Conclusion:**

A ten day training programme to write verbal autopsy report with adequate feed back sessions and random sampling of 5% of the verbal autopsy reports for re-interview worked very well in Tamilnadu, to arrive at the probable underlying cause of death reliably for deaths in early adult life or middle age (25–69 years) and less reliably for older ages (70+). Thus VA is practicable for deaths in early adult life or middle age and is of more limited value in old age.

## Background

In developed countries, data on disease-specific mortality by age are readily available from national vital registration. In developing countries, where 80% of the world's deaths occur, estimation of cause of death is more difficult because the levels of coverage of vital registration and reliability of cause of death stated on the death certificate are generally low (especially in rural areas).

A reliable assessment of disease-specific mortality rates is not yet possible in many parts of India, either because the underlying cause of the terminal illness was never known or because the relevant information was not recorded. For legal purposes death records do usually subdivide the causes of death into medical and non-medical (external) causes. But once non-medical causes have been excluded, specification of the underlying cause of a death from disease may be inaccurate, misclassified or missing for about 50% of adult deaths. For example, in Chennai, Tamilnadu, (south India) about half of those who died at home soon after the diagnosis of cancer (and whose deaths were therefore, in almost all cases, likely to have been caused by their cancer) do not have cancer mentioned on their death certificate [1], and for other diseases the problems might well be even worse.

Elsewhere, 'Verbal Autopsy' i.e., 'systematic retrospective inquiry of family members about the symptoms and signs of illness prior to death' has been used to help determine the underlying medical cause of death, particularly in childhood [2,3]. For childhood deaths in populations that are not covered by adequate medical services such "verbal autopsies" are now of established value in helping to classify the broad patterns of mortality. Verbal autopsies have also been used to assess the medical causes of maternal deaths [4-7]. Although in India there are about as many deaths in middle age as in childhood, there is less experience with verbal autopsies of adult deaths.

A special study on 'verbal autopsy' of adult deaths was conducted in urban and rural areas in the state of Tamil Nadu, south India during 1998–2000. The aims of the study were (a) to develop a verbal autopsy instrument and test its utility to determine the underlying cause of death and (b) to estimate cause specific mortality using underlying cause of death arrived based on verbal autopsy reports. Now we report the type of training given to the field interviewers to interview and write the verbal autopsy report for adult deaths, the procedure followed to arrive at the probable underlying cause of death, the accuracy of the instrument developed and change in the proportions of cause of death based on the underlying cause of death arrived by reviewing 80 000 verbal autopsy reports.

## Methods

### A: Training of field interviewers to write a 'Verbal autopsy' report

Male non-medical graduates with at least 15 years of formal education were selected. A ten days training was given on verbal autopsy interview techniques and writing verbal autopsy reports. There are four steps in training; 1). introduction to anatomy, signs and symptoms of various diseases, 2). mock interviews, 3). hands-on-training on writing verbal autopsy reports and 4). feed back session.

#### Step 1

This consisted of a basic three day introduction to anatomy, collecting data on history of past illness (refer Appendix I in Additional file 1), using symptoms/signs checklist of various diseases (refer Appendix II in Additional file 1) and to interview the surviving spouse/close associates or relatives of the deceased, the other members of the community such as neighbours to get data on train of events or circumstances preceding the death. Reports are to include complaints, symptoms, signs, duration of illness and treatment details of the illness prior to death.

The following data are to be ascertained (for all deaths due to medical causes) from the respondent to write the verbal autopsy narrative report:

• onset of illness prior to death: sudden or gradual

• major symptom(s) and associated symptom(s) – in chronological order

• If a symptom was present it was used as a filter to define what questions to be asked. For example, the filter symptom for heart attack was chest pain and the associated symptoms were breathlessness, sweating, vomiting and pain in the retrosternal area radiating to hand, shoulder, back etc. Cough for more than 4 weeks was a filter for lung cancer and tuberculosis. For each symptom, the duration should be recorded. Details of additional symptoms are built into the narrative in chronological order, by prompting, if necessary.

• progress of the illness

• any treatment received : Yes/No

• If yes, type of treatment received

• details of hospitalization prior to death:

○ name of the hospital (e.g. tuberculosis hospital, cancer hospital, coronary care unit etc),

○ duration of hospitalization,

○ whether discharged from the hospital against medical advice or not.

○ Status at the time of discharge from the hospital: alive/dead

• history of similar episodes and treatment(s) given

• abstract information related to the illness prior to death from the investigation reports done for any illness close to the time of death (within 6 months prior to the death) / hospital discharge summary etc, *if available*

• If a death certificate is available, copy the cause of death given on the death certificate (In the Tamilnadu study death certificates were available for only 20% of total deaths).

• While recording history of adults with long standing illness, the description should include details that occurred in the month preceding the death, with other information recorded in the past history section (Appendix I in Additional file 1).

• For deaths that occur during pregnancy, delivery, or within six weeks of delivery: use Appendix II (A and B) in Additional file 1

If the respondent is able to give the major symptoms and circumstances leading to death, then additional probing questions are asked about the associated symptoms using the symptoms/signs checklist (Appendix II(A & B) in Additional file 1) If the respondent is not able to give sufficient information on the symptoms of the illness prior to death or have difficulty in remembering any major symptom, then get necessary information to rule out non-medical causes of death. When the interviewer is sure that the death was not due to unnatural cause, the following procedure is used to collect necessary data on the symptom.

○ read out the filter symptom/sign of each module in the symptom/sign checklist

○ check responses to each, and note down positive responses

○ Where there is a positive response, additional details on that symptom and associated symptoms, if any, should be obtained.

Thus, the methodology of collecting data in the open format using 'symptoms/signs checklist' is an interactive process, with the respondent taking the lead in providing the information, and the interviewer prompting where necessary for more details. The Field Interviewer gathers as much information as possible on the underlying cause of death from the respondent. It is imperative to get a logical and complete history of symptoms, signs, events, investigations and treatment, so that the medical reviewer gets sufficient information to assign a probable specific underlying cause of death.

#### Step 2

In the following two days, mock interviews were organized to illustrate techniques of probing a respondent to get the required information on cause of death as well as how to write the verbal autopsy report in local language (Tamil) in Appendix I in Additional file 1 as stated by the respondent.

#### Step 3

The third component of training included three days of hands on verbal autopsy training in the field. To limit distress over the terminal event, the field visit was carried out at least six months after death. Name of the deceased, father's name (if the deceased was a male) or spouse name (if the deceased was a female), age, gender, informant's name and address of the deceased at the time of death were given to field interviewers to locate the house of the deceased. The Field Interviewers carry Appendix I and II (symptoms/signs checklist) in Additional file 1 to the field. They were blind to the cause of death stated on the death certificate. The Field Interviewer located the house of the deceased based on the data given to him. He introduced himself to the respondent and began the interview. Each one completed twenty reports which were reviewed and feedback was provided two days after completion of field work to maximize quality of writing the verbal autopsy report.

#### Step 4

The final component of training was feed back session for 2 days. This session involved teaching them how to include essential information in report writing. The feedback session mainly focused discussion on reports which did not have a specified underlying cause of death and reports with minimal information to arrive at the probable underlying cause of death; for example, a report may say that a person had a stroke ten days ago but did not specify the type of onset (sudden or gradual, whether the person was conscious or unconscious, had difficulty in speaking or not, which parts of the body may have been affected etc.) or a report may say that the deceased had fever for ten days and died. It did not give details about the fever and other associated symptoms if any.

### B: Verbal autopsy of 80 000 adult deaths (≥ 25 years at the time of death) in Tamilnadu, South India

This special verbal autopsy study was carried out in two areas in Tamilnadu. The Chennai city (urban) with a population of 4.2 million, and the Villupuram district (rural), with a population of 2.5 million were chosen for this study. We have successfully traced 48 000 adult deaths (≥ 25 years at the time of death) in urban area and 32 000 adult deaths in rural Villupuram district and reviewed 80 000 verbal autopsy reports to arrive at the probable underlying cause of death.

#### Mortality data in urban area (Chennai)

Information on deaths that occur in Chennai has been maintained manually by trained staff in Chennai Vital Statistics Department (VSD). The following data on deaths that occurred in Chennai during 1995 to 1997 were collected from the death registers in the Vital Statistics Department: deceased name, age, gender, marital status, father/spouse name, informant's name, occupation, place of death, address at the time of death, date of death and recorded cause of death (immediate, underlying and/or contributory). 72,000 deaths occurred during the study period of 1995–97. Of 72 000 found, 5000 deaths were attributed to external causes (unintentional injuries, suicide or homicide) in the death certificate, and were excluded. Of the remaining 67 000 deaths attributed in the VSD  to medical causes, 48 000 of the households were successfully visited during 1998–99 to try to assign cause of death by verbal autopsy.

#### Mortality data in rural area (Villupuram district)

All formal and informal village records were to be sought to identify all deaths at any age during 1997–98. 41,000 such records were identified and 39 000 of the households were successfully visited during 1999–2000 to try to assign cause of death by verbal autopsy. Of 39 000 deaths, 7000 were before age 25.

#### Feed back sessions and re-interview

Feed back sessions were organized regularly throughout the study period to improve the quality of the verbal autopsy reports and 5% of the field visit reports were validated by re-interview one week after completion of the main interview, and blind to its results. This re-interviewing was done by one or other of two special interviewers because knowledge that a resurvey might well take place would ensure reliably motivated fieldwork at the initial survey, and also to check whether there were any systematic defects in the technique of any of the field workers: none were found. The underlying cause of death arrived based on re-interview data was not substantially different from the one arrived based on main interview data.

#### Arriving at underlying cause of death

All verbal autopsy reports were centrally reviewed by two physicians independently in order to arrive at "probable underlying cause of death". Each made a diagnosis based on signs, symptoms and sequence of events prior to death given in the verbal autopsy report, which were then coded according to the 9^th ^International Classification of Diseases, Injuries and Causes of Death [8]. The same 2 physicians reviewed all the 80,000 verbal autopsy reports. The discrepancies in the underlying causes of death were noted in 5% of verbal autopsy reports. These were discussed and resolved. The disagreement between 2 physicians in arriving at underlying cause of death was noted before classifying causes of death into broad groups. For example, 'Pneumonia' and 'Lower respiratory infection' were grouped under 'Infection'. According to one physician the underlying cause of death was pneumonia and for another physician it was lower respiratory infection.

## Results

### Urban study

In Chennai city, the study was done in 1998–99 and the verbal autopsy reports were reviewed for 27 726 male deaths and 20 631 female deaths. Table 1 shows about 1100 (M:683, F:456) deaths due to medical causes were reassigned to external causes based on verbal autopsy reports. Deaths from unspecified medical causes and unknown causes decreased from 54% to 23% (p < 0.0001).

**Table 1:** Cause of death from Vital Statistics Department* and based on Verbal Autopsy of 48 000 adult deaths (aged ≥ 25) in Chennai (urban), south India: 1995–97

Causes of death (ICD9 codes)	Cause of death in VSD		Cause of death based on Verbal Autopsy	
	M (%)	F (%)	M (%)	F (%)
Vascular disease (390–415, 418–459)	8319 (30)	5168 (25)	11056 (41)	7435 (37)
Respiratory tuberculosis (TB) (011, 012, 018)	1399 (5)	372 (2)	2231 (8)	575 (3)
Other respiratory diseases (416, 417, 460–519)	1088 (4)	596 (3)	1597 (6)	855 (4)
Neoplasm (140–239)	1163 (4)	1002 (5)	2344 (9)	1999 (10)
Infection except respiratory & TB (rest of 1–139, 279.8 [HIV], 320-6, 590, 680-6)	584 (2)	303 (2)	1034 (4)	618 (3)
Unspecified medical causes (780-9, 797-9)	12291 (44)	115 11 (56)	4367 (16)	5889 (29)
Other specified medical causes	1899 (7)	1045 (5)	4414 (16)	2804 (14)
No cause given in VSD (hence probably medical)	983 (4)	634 (3)	Nil	Nil
Total deaths – medical	27 726	20 631	27 043	20 175
Re-assigned by VA to external causes	*Excluded from the study		683	456
Total deaths (medical causes+external causes)	27 726	20 631	27 726	20 631

*Deaths(M: 3644; F:1644) that were assigned by the Vital Statistics Department(VSD) to non-medical causes were excluded from the study

### Rural study

In Villupuram district, verbal autopsy report was written for all deaths i.e., deaths due to medical and external causes. So verbal autopsy reports of 19 294 male deaths and 12 494 female deaths were reviewed. Deaths from unspecified medical causes and unknown causes decreased from 41% to 26% (p < 0.0001) (Table 2).

**Table 2:** Cause of death from various local records in Villupuram district and based on Verbal Autopsy of 32 000 adult deaths (aged ≥ 25) in Villupuram (rural), south India: 1997–98

Causes of death (ICD9 codes)	Cause of death in local records		Cause of death based on Verbal Autopsy	
	M (%)	F (%)	M (%)	F (%)
Vascular disease (390–415, 418–459)	3351 (20.3)	1614 (14.4)	3928 (24.6)	2404 (22.0)
Respiratory tuberculosis (TB) (011, 012, 018)	1659 (10.1)	686 (6.1)	1841 (11.5)	671 (6.1)
Other respiratory diseases (416, 417, 460–519)	717 (4.4)	471 (4.2)	1044 (6.5)	728 (6.6)
Neoplasm (140–239)	415 (2.5)	594 (5.3)	488 (3.1)	664 (6.1)
Infection except respiratory & TB (rest of 1–139, 279.8 [HIV], 320-6, 590, 680-6)	1818 (11.0)	1584 (14.1)	1954 (12.2)	1411 (12.9)
Unspecified medical causes (780-9, 797-9)	5829 (35.4)	4565 (40.7)	4173 (26.1)	2737 (25.0)
Other specified medical causes	2237 (13.6)	1346 (12.0)	2570 (16.1)	2334 (21.3)
No cause given (hence probably medical)	451 (2.7)	343 (3.1)	Nil	Nil
Total deaths – medical	16 477	11 203	15 998	10 949
External causes	2817	1291	3296	1545
Total deaths (medical causes+external causes)	19 294	12 494	19 294	12 494

### Validity of verbal autopsy tool

The cause of death stated on the death certificate is often inaccurate. Studies, which have been undertaken around the world, show substantial difference (10–40%) between the clinical diagnosis or clinical cause of death and postmortem findings [9-12] and many of the completed death certificates failed to provide relevant information to allow adequate ICD-10 coding [13]. In India, individuals whose deaths might have been due to external causes are often subjected to postmortem examination, but others are not. So it is not possible to compare (clinical diagnosis of) medical causes of death against postmortem findings in India. Gajalakshmi et al [1] had done a study in Chennai to determine the sensitivity of the death certificate to identify cancer by comparing the cause of death stated on the death certificate with the morbidity date base of Chennai population-based cancer registry. It was found that the sensitivity of the death certificate to identify cancer as the underlying cause of death was 57%. In Chennai, about 75–80% of cancer patients attend health care facilities at late stage of the disease; about half of those who died at home soon after the diagnosis of cancer (and whose deaths were therefore, in almost all cases, likely to have been caused by their cancer) did not have cancer mentioned on their death certificates. Hence verbal autopsy tool was developed to determine specific cause of death, to compute cause specific death rates.

Where a cause recorded on the death certificate in the VSD differed from the underlying cause assigned by the VA, there was often no absolute way of knowing which was correct (where the assigned cause by a medical doctor on the death certificate lacked detail, the VA may well be more reliable, and vice-versa) except for cancer deaths which could be verified with the Chennai cancer registry records. Hence, the validity of VA diagnosis was assessed only for cancer deaths(ICD 9:140–208) by comparing with the stated cause of death in the VSD records and verifying with the Chennai cancer registry records and hospital medical records (only for cancer diagnosis). Chennai Population-Based Cancer Registry is a demographic registry in the network of the Indian Council of Medical Research, Govt. of India and has been functioning since 1982 at the Cancer Institute(WIA), Chennai. Cancer is not a notifiable disease in India. Hence registration has been done by active method. Cancer patients attending the Govt. hospitals are interviewed to collect data on age, sex, address, duration of stay in Chennai city, marital status, mother tongue and educational level. Interviews are done at the houses for those who have been missed by the registry staff during their (Govt.) hospital visit. The clinical data, such as date of cancer diagnosis, method of diagnosis, site of cancer, any spread of the disease, histology, treatment details and status (alive or dead, if dead at the hospital, then, date and cause of death) for all registered patients are abstracted from the hospital medical records. All data on cancer patients attending the private hospitals are abstracted from the hospital records. The mortality data available at the Vital Statistics Department are linked with the Chennai Cancer Registry data base. Therefore the cause of death arrived based on verbal autopsy report was compared with the hospital data on cancer patients available in the Chennai Cancer Registry data base.

Table 3 shows that 3053 deaths were identified as being due to cancer by VA. Review of VSD records revealed that 1435 of 3053 deaths as being due to non-cancer causes (majority of deaths were attributed to ill-defined/unknown followed by vascular causes) and 1618 (of 3053 deaths) as being due to cancer. Since the sensitivity of death certificate to identify cancer as underlying cause of death is only 57% in Chennai [1], all deaths at ages 25–69 included in the present study were verified with Chennai population-based cancer registry records. Table 4 shows that out of 3053 deaths identified by VA as cancer underlying cause of death, 2765 deaths matched with Chennai population-based cancer registry data base and 288 deaths did not. The cancer deaths identified by VSD records and not by VA (n = 107) (Table 3) matched with Chennai population-based cancer registry data base. Thus 288 cancer deaths, identified by VA, were not registered in the Chennai population-based cancer registry [14,15]. These were missed by the Chennai population-based cancer registry, both in the routine morbidity and mortality data registration process. We were successful in identifying all 288 cancer deaths, not available in the Chennai population-based cancer registry, in the medical records of the hospitals located in Chennai city. Thus all 3053 cancer deaths identified by VA were confirmed by linking with Chennai Cancer Registry records and hospital medical records. So there were no false positive cancer deaths recorded by VA. The sensitivity of VA to identify cancer was 94% (1618/1725) compared to VSD records and 96% (2765/2872) compared to Chennai population-based cancer registry in the age group 25–69 [15,16] and the Chennai population-based cancer registry missed 9% (288/3160) of total cancer deaths in the early adult life and middle age during the study period of three years.

**Table 3:** Cancer (ICD 9: 140–208) deaths at ages 25–69 by Verbal autopsy (VA) and in Vital Statistics Department records (VSD) in Chennai (urban), South India

	VSD			
	
VA		Cancer	Noncancer	Total
Cancer		1618	1435	3053
Noncancer		107	21941	22048
Total		1725	23376	25101

**Table 4:** Cancer (ICD 9: 140–208) deaths at ages 25–69 by Verbal autopsy (VA) and in Chennai population-based cancer registry in Chennai (urban), South India

	Cancer Registry			
	
VA		Registered	Not registered	Total
Cancer		2765	288	3053
Noncancer		107	21941	22048
Total		2872	22229	25101

## Discussion

The Tamilnadu study on verbal autopsy [15,16] used university graduates since it is very expensive to send professionally trained individuals to field visits, to write verbal autopsy reports. We have found it very difficult to get female graduates willing to do field work. Hence only males were recruited for the field work. Responders of female deaths were usually males who did not hesitate to reveal the circumstances/ symptoms etc prior to death. The participation rate was 100%. The informants were given full information about the objectives of the study and the participation in this study was entirely voluntary basis.

The verbal autopsy tool for adult deaths is an open narrative format uses the check list of symptoms and signs with filters to get more information on train of events or circumstances preceding the death. The sensitivity of this tool is 95% (94% compared to VSD records and 96% compared to Chennai population-based cancer registry) in the age group 25–69 during the study period. The validity of this verbal autopsy tool is influenced by the training given to the interviewers, on the immediate random checking of the 5% of interview data and reviewing of the field reports centrally by 2 physicians to arrive at the probable underlying cause of death which is better than that arrived by opinion-based algorithm [17]. There is little information or literature about validity of cause of death for adults by verbal autopsy in India.

As a result of using verbal autopsy method, adult deaths (≥ 25) from unspecified and unknown causes decreased from 54% to 23% (p < 0.0001) in urban and from 41% to 26% (p < 0.0001) in rural areas in Tamilnadu. Ten day training to write verbal autopsy reports followed by constant monitoring of the submitted reports resulted in arriving at the probable underlying cause of death for most of the deaths and to compute broad classification of the underlying causes of about 90% of deaths in early adult life or middle age: in old age, however, the proportion classifiable is substantially lower. The specific causes of death arrived based on verbal autopsy reports were used to estimate death rates for Chennai city [16] and to estimate the risk of death associated with smoking for broad groups of causes of death[18].

This methodology of arriving cause of death for adult deaths by verbal autopsy is now being adopted by the Registrar General of India, Govt. of India, for nationwide use in the Sample Registration System(SRS) that consists of 6671 units (4436 rural and 2235 urban) spread across the country covering 1.1 million households and about 6 million population. The SRS is a large demographic survey of vital events occurring in a national random sample of urban and rural areas in India by the Registrar General of India (RGI) to provide annual estimates of age-specific birth and death rates at the national and state levels. This exercise on verbal autopsy for adult deaths along with the verbal autopsy questionnaire developed by the SRS collaborators for deaths that occur at ages less than 15 years and for maternal deaths is expected to yield reliable cause-specific death rates for India.

## Conclusion

A ten day training programme to write verbal autopsy report with adequate feed back sessions and random sampling of 5% of the verbal autopsy reports for re-interview worked very well in Tamilnadu, to arrive at the probable underlying cause of death reliably for deaths in early adult life or middle age (25–69 years) and less reliably for older ages (70+). Our experience shows that the open narrative, if well written, provides adequate information for assigning probable underlying cause of death for adult deaths.

## Competing interests

The authors declare that they have no competing interests.

## Authors' contributions

VG and RP participated in designing the study, analysis and preparation of the report. Development of verbal autopsy tool, training on verbal autopsy method, co-ordination of field work and data management were done by VG. Verbal autopsy review by VG and TS Kanaka, physician.

## Pre-publication history

The pre-publication history for this paper can be accessed here:



## Supplementary Material

Additional File 1 Appendix I – used to write the history of past illness and verbal autopsy report for adult deaths (25 years or older) and in Appendix II – Symptoms/signs checklist for adult deaths (≥ 25 years) is given Click here for file
