# Supplementary material for: Verbal autopsy of 80,000 adult deaths in Tamilnadu, South India
Source: BMC Public Health. 2004 Oct 15;4:47. doi: 10.1186/1471-2458-4-47 (PMC535898; doi:10.1186/1471-2458-4-47)
Supplement: Additional File 1 — Appendix I – used to write the history of past illness and verbal autopsy report for adult deaths (25 years or older) and in Appendix II – Symptoms/signs checklist for adult deaths (≥ 25 years) is given [file 1471-2458-4-47-S1.doc]

# Appendix I

**ADULT DEATHS (25 YEARS OR OLDER**)

**Serial No.__________**

**Name of the deceased____________________ _Age_____ Sex_____**

The following questions represent the most common past medical conditions present among adults. The answers to all these questions may assist in arriving at the probable underlying cause of death.

| **History of past illnesses, prior to death. Code: yes-1, no-2, unknown-9** | | | |
| --- | --- | --- | --- |
| 1. Hypertension | 1 2 9 | 6. HIV/AIDS | 1 2 9 |
| 2. Heart disease | 1 2 9 | 7. Cancer, specify Site_________ | 1 2 9 |
| 3. Stroke | 1 2 9 | 8. Asthma | 1 2 9 |
| 4. Diabetes | 1 2 9 | 9. Injury history | 1 2 9 |
| 5. TB | 1 2 9 | 10. Other chronic illness, Specify______________ | |

**Verbal autopsy report:**

**Appendix II**

**A: SYMPTOMS/SIGNS CHECKLIST FOR ADULT DEATHS (≥25 YEARS)**

Use this checklist for probing the details of illness so as to write the verbal autopsy report.

The checklist consists of two sections: deaths attributed to non-medical causes, and those due to illnesses. The list has 9 main symptoms or conditions called lead symptom or filter symptom. The main symptom may appear as an associated symptom for another main symptom. For example breathlessness or difficulty in breathing and cough are main symptoms for ‘respiratory illnesses’ and breathlessness is an associated symptom for ‘heart attack’. Fever is the main symptom for ‘viral infections’ and is an associated symptom for ‘respiratory illnesses’.

1. **Non-Medical causes: unintentional or intentional**

- Road traffic injuries related to any motorized or non-motorized modes of transport
- Falls at home or any other pace, fall of objects, burns (including hot objects, vessels or electrical injuries), drowning (include from floods), poisoning (accidental or intentional), bite/sting (mention name of the insect/animal), natural disaster (lightning, sunstroke, flood, earthquake,)
- Fracture: write details of where, when, how
- Suicide (may need to ask neighbours or others to obtain this history)
- Homicide (may need to ask neighbours)
- Enter the time interval between the accident and death
- Did the person die at the site of accident? If not, for how many days did he/she live?
- Type of medical care, and where received
- Any chronic conditions/illness in the month prior to injury death?

1. **SYMPTOMS/SIGNS OF ILLNESSES:**

**1.Symptom: chest pain**

- Ask about onset (sudden or gradual), duration of pain (pain lasted for more than 24 hours or less than 24 hours)
- Location of pain (chest, upper abdominal, etc)
- Spread of pain to any part of the body, e.g., pain in behind the central part of chest (retrosternal), hand, shoulder, back etc.
- Whether pain was increased by cough/deep breath, or by touching the area or walking/after eating
- Associated symptoms: breathlessness, sweating, vomiting, loss of consciousness
- Write history from childhood till death: any history of fever with joint pain and swelling, chest pain or heart attack and any treatment, medication in detail, including surgeries.

**2. Symptoms: Cough and or Breathlessness**

**2.1 Cough**

- Cough: dry, productive (with sputum) or with blood (haemoptysis),
- Severe bouts of cough with whoop at the end, cough only at night, enquire when cough was worse (day or night)
- Always sitting in bed for relief of cough
- Localized pain (pain at the sides of the chest wall increased by cough and/or deep breath)

**2.2** **Breathlessness**

- Write details of onset and progression of breathlessness {e.g. breathlessness is initially on exertion (i.e., not present at rest), but progressively worsens to a stage of breathlessness even at rest}
- Breathlessness occurring soon after lying flat and relieved by sitting up
- Breathlessness at rest, triggered by allergy or chest infection
- Episodes or attacks of wheeze and breathlessness of sudden onset (may be triggered by allergy or chest infections)
- May be accompanied by swelling of hands and legs, generalized swelling of the body, enlarged abdominal swelling or fluids in chest

**2.3** **Other Symptoms associated with cough, breathlessness**

- Weight loss
- Hoarseness of voice
- Night sweats
- Evening rise of temperature
- Fever with generalized aches and pains
- Vomiting

**3. Fever**

- High grade/low grade fever for how many days?
- Continuous with no normal temperature, intermittent (on and off) fever, or occasional
- Repeated attacks of fever with chills, rigor (shaking), sweating, myalgia (muscle pain)
- High fever followed by skin eruption (rash/blisters) the next day
- Fits, confusion, drowsiness, coma
- Associated with cold, dry cough, headache, generalized ache
- Coated tongue, jaundice, diarrhoea, burning sensation while passing urine, chest pain, neck stiffness, irritated and does not like light, sound etc
- Fever for more than 30 days: **refer to symptoms for HIV/AIDS**

**4. Symptom: Paralysis**

- Was paralyses accompanied by sudden loss of consciousness?
- Time of onset: during activity or in sleep
- Progression: over minutes, hours or noticed on waking up with or without vomiting/ headache
- Note if paralysis in any part of the body in the month preceding death
- Write affected part of the body: half of the body, one upper limb– right/left side, face, loss of speech, lower limbs
- Was it associated with loss of memory, loss of vision, altered speech, loss of urinary control, loss of sensation of any part of body, Other features: convulsions (fits), neck stiffness, giddiness, hypertension

**5.Symptom: Seizures/ fits**

- History of sudden jerky movements of limbs with or without loss of consciousness accompanied by rolling of eye balls and frothing of mouth, with loss of consciousness, loss of memory, awake between convulsions or not, tongue bite, bed wetting, confused
- History of head injury
- Type and duration of treatment taken etc.

**6. Symptom: difficulty in passing urine or low urine out put**

- Abrupt onset with puffiness of face or swelling of eyelids in the morning, low urine amount, passing urine with pus, passing urine with blood, localized/generalised swelling of hands and legs, swelling or fluid in the abdomen and/or fluid in the chest
- Frequent passage of urine, pain in middle of lower abdomen, intense desire to pass more urine even after the bladder has been emptied
- Tenderness in the side of abdomen, sudden onset of pain in one or both loins, spreading to lateral part of lower abdomen and above genital area
- Pallor, nausea, vomiting
- Become dull, drowsy, coma (unconsciousness) and death
- History of kidney transplantation
- History of high blood pressure

**7.Sign: Oedema (swelling of feet and hands or body)**

- Did she/he look pale?
- Loss of weight
- Presence of breathlessness at rest; aggravated by walking (refer symptoms under 2.2)
- Fatigue, feeling the heart beat faster, nausea, loss of appetite
- Generalized swelling of feet and hands

1. **Symptoms related to abdomen/GI tract**

**8.1: abdominal pain or swelling**

- Abdominal pain: localized or generalized, type (sharp, dull, throbbing, continuous) and relationship to food intake (pain was more on empty stomach and relieved after taking food or pain increased after taking food)
- Abdominal distension : sudden or gradual
- Other symptoms: loss of appetite, nausea, constipation, black stools, vomiting with blood, breathlessness and sweating with sudden abdominal pain
- History of surgery or trauma or cancer
- Lump/mass in abdomen
- Difficulty in swallowing: solid/liquid food

**8.2 Symptom: stomach pain/ulcers**

- Had peptic ulcer: burning pain, localized to middle part of upper abdomen or extending to chest, recurrent abdominal pain
- Typically pain wakes the patient from sleep around 2 AM and is relieved by food, milk, antacids, belching or vomiting
- Periodicity: pain occurs in episodes, lasting 1-3 weeks every time, 3-4 times per year
- Natural history of spontaneous re-occurrence and free from symptom lasting for decades or even life
- Relationship to food: pain occurs on empty stomach (hunger pain) and is relieved by food or antacids
- Vomited blood, had been drinking alcohol
- Other symptoms: loss of appetite, nausea

**8.3 Symptom: diarrhoea/dysentery or blood in stools**

- Loose/semisolid stools, blood /mucus in stools, watery/rice water like stools, Painless profuse (large quantity) diarrhoea
- How many times a day at worst?
- Vomiting, excessive thirst and dehydration (less water in the body: sunken eyes, diminished urine amount, dried tongue) ,fever ( sudden onset).
- Blood in the stool, colour red or black. Any history of cancer?
- Did the deceased have food in any party or any gathering few days prior to the event of diarrhoea? If yes, did any other person who had food from the same party also suffer from loose motion?

**8.4. Symptom: Jaundice (yellowness in the white part of eyes)**

- Eye/skin colour change to yellow, urine also dark yellow in colour
- Marked swollen, bloated abdomen with swelling of feet and then face and hands
- Vomiting blood, history of drinking alcohol regularly
- Any history of cancer

**8.5. Symptom: Local swelling in the groin/scrotum:**

- History of reducible swelling in scrotum (used to appear on coughing or straining and then disappeared)
- Able to push back the swelling with out pain
- Became painful, tender and not able to reduce before death

1. **Symptoms/signs of various illnesses:**

**9.1. HIV/AIDS:**

- Loss of weight and degree (percentage) of weight loss (this is a key symptom)
- Any ulcers or sores in the genital area (sexually transmitted infection or venereal disease)
- Fever for more than 30 days
- Diarrhoea for more than 30 days
- Persistent cough for more than 30 days
- Generalised generalized swelling of nodes in arm pits, neck, groin
- Generalised itching and skin rash
- Did she/he have white sores in mouth (white patches)?
- Did she/he have any skin disease?
- Had multiple sexual partners
- Had been injecting drugs
- Any test done to confirm HIV/AIDS. If so, write name of the facility and when was it done
- Had any one in the family (spouse or parent) has HIV/AIDS
- Had TB (For symptoms - refer above under Respiratory tuberculosis)
  1. **Tetanus:**
- Locked jaw (unable to open mouth), history of stiffness of neck/back of the body and fits
- History of open wound, animal bite/sting, burns, fracture, fireworks
- Injury, bad wound in limb
  1. **Leprosy:**
- History of unhealed ulcers
- Disfigurement
  1. **Mental Disorders**
- History of sudden changes in behaviour, sudden spells of excessive crying, isolation, withdrawn nature
- History of aggressive, unusual or violent behaviour
- History of excessive talking, incoherent or self talking
- Reporting hearing of voices
- Passing into sudden bouts of unconsciousness
- Loss of memory and difficulty in recalling names of objects or persons

**9.5 Diabetes:** Also known as sugar disease. May have the history of:

- Increased appetite, thirst, increased frequency of urination
- Weight loss / weight gain
- Unhealed ulcer, amputation
- Gangrene (blackening of the skin due to serious and permanent arterial obstruction)
- Diabetic coma (unconsciousness)

**9.7 Cancer**

- Loss of weight
- Lumps or ulcers rapidly increasing in size over the period
- Difficulty in swallowing or breathlessness for more than a month
- Prolonged cough
- Loss of appetite
- Bleeding from various body openings (eg. Bleeding PV)
- History of taking treatment for cancer.
- Write the site of the cancer, type of treatment received and details on spread of cancer, if possible, as stated by the respondent.

**B: MATERNAL DEATHS**

Maternal deaths – include deaths during pregnancy, delivery, or within six weeks (42 days) of delivery or abortion.

Collect the following information on delivery:

Duration of pregnancy, history of antenatal care, type of delivery (normal, caesarean, forceps or vacuum), complications of pregnancy (prolonged labour, difficulty in delivering the placenta, fits, loss of consciousness, hypertension, excessive bleeding - in the beginning of labour pains or during labour or after delivery), history of puerperal infection/sepsis, date of delivery, place of delivery- domiciliary or iInstitutional.

Collect the following information on abortion:

Spontaneous or induced, place of abortion- domiciliary or iInstitutional.

Who attended it? Doctor, Midwife/nurse or traditional birth attendant
